# Issues in matching learners to their reading levels using receptive vocabulary knowledge – A closer look at the vocabulary levels tests and scoring methods

**DOI:** 10.1016/j.heliyon.2022.e10244

**Published:** 2022-08-18

**Authors:** Hung Tan Ha

**Affiliations:** School of Foreign Languages, University of Economics Ho Chi Minh City (UEH), Ho Chi Minh City, Viet Nam

**Keywords:** Vocabulary levels test, Vocabulary size, Reading comprehension, Cut-off score

## Abstract

The vocabulary levels tests (VLT) are very powerful instruments for the measurement of learners' vocabulary knowledge and have been widely used by language teachers in the task of matching learners to their reading materials. However, their accompanying score interpretation methods have not been backed by research and concerns have been raised regarding these scoring methods' appropriateness. The study began by looking at the VLTs and their scoring methods since the day they were born and then went on to investigate potential issues in the scoring methods that have been used together with the VLTs: the cut-off score and vocabulary size score. Tests of receptive vocabulary knowledge and English reading proficiency were administered to 234 university students. Research findings problematized the application of VLTs and cut-off scores on the ranking of large groups of students and supported the use of vocabulary size score as a more suitable method for vocabulary knowledge research. Implications for teaching and learning were also provided.

## 1 Introduction

Vocabulary is one of the most important element in language learning (Nation, 2013). Over decades, researchers in the field have built a strong found foundation regarding the relationship between vocabulary knowledge and comprehension (Nurmukhamedov and Webb, 2019). The aim of these studies was to tell people how well vocabulary knowledge correlated with language proficiency (Ha, 2021b; Cheng and Matthews, 2018; Matthews and Cheng, 2015; Qian and Lin, 2020; Schmitt et al., 2011; Stæhr, 2009; Lange and Matthews, 2020; van Zeeland and Schmitt, 2013) and how many words English learners would need to comprehend certain types of texts (Al-Surmi, 2014; Dang and Webb, 2014; Nation, 2006; Nurmukhamedov, 2017; Nurmukhamedov and Sharakhimov, 2021; Tegge, 2017; Webb and Macalister, 2013; Webb and Rodgers, 2009a, 2009b).

To serve the purpose of estimating learners' vocabulary knowledge, several measuring instruments have been created, including the Vocabulary Size Test (Nation and Beglar, 2007) and Vocabulary Levels Tests (Ha, 2021a; Schmitt et al., 2001; McLean and Kramer, 2015; Webb et al., 2017). Along with the development of vocabulary tests, the accompanying scoring methods were also put forward by the test creators themselves. And for many years, researchers have use these vocabulary tests in accordance with these suggestions without controversy.

Recently, a debate between top scholars in the field (Laufer, 2021; McLean, 2021; McLean and Stoeckel, 2021) on the scoring method of vocabulary levels tests really made vocabulary linguists reconsider the practice they have been following for decades. The main topic of the discussion was on the relationship between vocabulary level/ size and reading comprehension. On the one hand, McLean (2021) and McLean and Stoeckel (2021) criticized the old cut-off scores, which were primarily based on personal opinions and speculations, and called for an evidence-based threshold for mastery levels. On the other hand, Laufer (2021) believed that setting a cutting-score for vocabulary tests was not at all necessary and that the use of vocabulary size score worked well for most purposes. In this paper, the relationship between vocabulary knowledge and reading comprehension will be revisited in the lens of both vocabulary level and size.

## 2 Literature review

### 2.1 A brief history of vocabulary levels tests

The Vocabulary Levels Test (VLT) was originally created by Nation in 1983 as a diagnostic test to determine whether learners had achieved mastery of words at different frequency levels as well as academic words. Nearly 20 years later, in 2001, Schmitt, Schmitt and Clapham introduced an improved version of the VLT and carried out a validation study. This could be seen as the first validity evidence of the VLT. Schmitt et al.’s (2001) VLT employs a matching format in which the words are presented in clusters of six words and three definitions. With ten clusters containing 30 definitions at each level, the test assesses 150 target words at four word-frequency levels (2000, 3000, 5000, 10,000) and an academic vocabulary level. The test became one of the most popular tool for estimating vocabulary knowledge for over a decade later.

In response to the major concerns raised by Webb and Sasao (2013) regarding the VLT’s outdated test items as well as its inability to capture vocabulary knowledge at the 1000 and 4000 levels. Webb et al. (2017) introduced two parallel forms of the Updated Vocabulary Levels Test (UVLT). The UVLT generally inherits the VLT’s matching format with 10 3-item clusters consisting of 15 nouns, 9 adjectives and 6 verbs per word level. Besides using the Nation’s (2017) up-to-date BNC/COCA word list, Webb et al. (2017) excluded the 10,000 frequency level and the academic word level, replacing them by two words levels measuring the 1000 and 4000 word-frequency levels. The 5-level, 150-item test is believed to reflect a shift toward higher-frequency word levels (Stoeckel et al., 2021).

While Webb et al. (2017) held that the assessment of the AWL should not be incorporated in a vocabulary levels test together with other word-frequency levels in the BNC/COCA, not every linguist shared the same perspective. In 2015, McLean and Kramer published a New Vocabulary Levels Test (NVLT) which also contained 150 test items and measured knowledge of the first five 1000-word levels in the BNC/COCA list (Nation, 2017). Still, the NVLT had some critical differences compared to the UVLT. The first one was that the test employed the 4-option, multiple-choice format of Nation and Beglar’s (2007) Vocabulary Size Test. Secondly, McLean and Kramer’s (2015) NVLT also measured knowledge of the AWL together with the first five word levels in the BNC/COCA word list. Since their test measured six word levels but did not contain more test items than the UVLT, as a result, the 1000, 2000, 3000, 4000 and 5000 word levels in the test only contained 24 item each. The number of test items at the academic word level remained 30. The test’s options were later translated into Japanese to create a bilingual form (McLean and Kramer, 2016).

In an attempt to create a test of phonological vocabulary knowledge, McLean et al. (2015) created the Listening Vocabulary Levels Test (LVLT) and conducted a preliminary validation study. It could be said that the LVLT is a spoken version of the bilingual NVLT as the only difference between the two tests is that the target words and the context defining sentences were in spoken form. 6 years later, Ha (2021a) translated the test items into Vietnamese, created another bilingual version of the test and carried out another validation study of the test in another context.

### 2.2 Vocabulary levels tests and the accompanied scoring methods

It is worth noting that the purpose for VLTs to be created was to tell language teachers where vocabulary learning should be focused (Nation, 1983, 1990, 2008; Nation and Webb, 2011; Read, 2000; Webb and Sasao, 2013; Webb and Nation, 2017; Webb et al., 2017; Webb, 2021). And the cut-off scores were put forward for that sole purpose (Schmitt et al., 2001). However, despite being born for research, the cut-off scores were “not borne out by research” (Laufer, 2021, p. 241). In fact, vocabulary levels test creators proposed the cut-off scores for their VLTs based on the belief that such cutting scores would work well for their tests in particular situations (McLean and Kramer, 2015; McLean et al., 2015; Schmitt et al., 2001; Webb et al., 2017). That is exactly the reason why these thresholds for mastery are being challenged for their validity (McLean, 2021; McLean and Stoeckel, 2021).

Some researchers, with or without paying attention to the suggestion of VLTs creators, have utilized the VLTs together with their cut-off scores to examine the relationship between vocabulary knowledge and comprehension (Stæhr, 2009; Noreillie et al., 2018) or to report on the vocabulary knowledge of learners in particular countries (Dang, 2020; Webb and Chang, 2012). Most studies tried to use the cut-off score in combination with VLTs had the tendency to rank their participants to certain vocabulary levels, rather than look at the participants' overall performance. This means that test takers can only belong to one particular vocabulary level, despite the fact that they may have achieved the mastery of different levels. In one of the most cited studies, Stæhr (2009) applied a cut-off score of 27/30 and classified participants into 4 levels: “mastered only the 2000 vocabulary level”, “mastered the 2000 and 3000 levels” and “mastered the 2000, 3000 and 5000 levels” and “mastered all four vocabulary levels” (pp. 593–594). The mean results of participants' performance on a listening test were then estimated and compared against the lexical demands of the listening transcripts to examine the amount of words needed for reasonable listening comprehension (Stæhr, 2009).

Other researchers used vocabulary score, a scoring method designed for the estimation of vocabulary size, in combination with VLTs for their research (Ha, 2021b; Laufer and Ravenhorst-Kalovski, 2010). In their study, Laufer and Ravenhorst-Kalovski (2010) viewed each correct answer on the VLT as a representative of 5000/150 = 33.33 word families. Laufer and Ravenhorst-Kalovski (2010) multiplied students' scores on the VLT by 33.33 to calculated their vocabulary size and then ranked the participants to different vocabulary size group in accordance with their vocabulary knowledge. For example, students with vocabulary scores from 500 to 1500 were placed to the 1000 group. Those who knew 1500–2500 word families were then placed into the 2000 group. Participants with a score representing 2500–3500 word families were placed in the 3000 group. The 4000 group was for those who had score ranging from 3500 to 4500. And participants with knowledge above 4500 word families were placed in the 5000 group. This investigation into the relationship between vocabulary knowledge and reading comprehension has been one of the most influential in the field and its findings got cited in countless studies.

### 2.3 Issues in the two scoring methods and the indication of learners' reading level

As McLean (2021) emphasizes, the indication of learners' vocabulary knowledge is crucial to the selection of appropriate reading materials. For example, according to Nation (2007), language-focused or form-focused instructions would require a lexical coverage of no less than 85% (Stoeckel et al., 2021; Schmitt et al., 2011). For supported reading comprehension, learners would need to be familiar with 95% of the running words in their reading texts (Laufer, 1989; Schmitt et al., 2011). If the lesson involves meaning-focused or extensive reading, learners would be expected to know 98% of the tokens in their reading materials (Nation, 2007; Webb and Nation, 2017). Fluency development or speed reading materials would require a 100% coverage threshold (Nation, 2007).

The only limitation of vocabulary score is believed to be its inability to support English teachers in the task of matching reading materials to learners' vocabulary mastery level, which would in turn facilitate suitable teaching practice (McLean, 2021; McLean and Stoeckel, 2021). It is also held that students with the same vocabulary size may have different levels of vocabulary mastery and language proficiency (Stoeckel and Bennett, 2015). However, Laufer and Aviad-Levitzky (2017) showed that the vocabulary size score could match learners to their reading level with the accuracy as high as 91%.

Compared to the vocabulary score, more concerns have been raised regarding the other scoring approach, the cut-off scores. The major issue lies with the ranking nature of the score interpretation method. According to Schmitt et al. (2001), learners who mastered the lower-frequency levels were assumed to have mastered all the higher frequency levels. However, we all know that this cannot always be the case. Assuming a score of 27/30 is the threshold for vocabulary mastery, then learners who scored 27/30 at the 4000 and 5000 levels for the UVLT but scored 26 or less at the 3000 level would be good examples. Since a participant can belong to only one vocabulary level (Dang, 2020; Stæhr, 2009), therefore, in principles, researchers usually have three options to choose from when facing this situation. The first one is to leniently place these students at the highest level that they reached their mastery, in this case, the 5000 level. The second option is to strictly place these participants at the level right below the level where they missed their criterion, in this case, the 2000 level. The third way is to safely exclude these participant from data analysis, and only report data from the rest.

From my point of view, except the third option, picking any strategies mentioned above would be problematic. The first option would violate the hypotheses proposed by Schmitt et al. (2001) and Webb et al. (2017) as we were accepting that certain learners placed at 4000 or even 5000 levels failed to mastered the higher frequency levels. The second choice would rank learners at the levels which would not be able to represent their vocabulary knowledge and language proficiency, and therefore potentially falsify the concept of mastery at a certain word level. Even when such hypotheses are not in evidence, a 27/30 cut-off score would still rank students who scored 30-26-25-22-20 and 29-15-10-0-1 as the being same level. As a consequence, multi-level gaps could be created between students with 1 or 2 scores differences, which is unfair and even misleading (Ha, 2021b; Laufer, 2021).

This method of ranking may provide a false image of learners' vocabulary knowledge and language proficiency at a certain vocabulary level, which could therefore raise further concerns on the validity of the findings. It is worth noting that this problem is especially common when we apply a general cut-off score on a large group of test takers to rank them according to their performance on the VLTs. And it is even more notable that the practice has been welcomed with open arms by researchers in the field (Dang, 2020; Noreillie et al., 2018; Stæhr, 2009). However, an evidence-based answer on whether or not such an issue should really be the cause for concern has not been provided to date.

### 2.4 The present study

The present study was carried out to investigate whether cut-off scores would rank students of various vocabulary sizes at the same vocabulary level. And more importantly, if receptive vocabulary knowledge was found to have strong correlations with reading proficiency (Ha, 2021b; Laufer and Ravenhorst-Kalovski, 2010; Qian, 2002; Stæhr, 2008), then this ranking would also lead to the classification of learners of significantly different reading proficiency.

Moreover, as Laufer has so much confidence in the vocabulary size score and its ability to match students to their reading level (Laufer and Aviad-Levitzky, 2017; Laufer, 2021), then it is hypothesized that learners of the same vocabulary size group would have similar reading proficiency, regardless of whether they have or have not satisfied the requirements of particular mastery threshold.

The research aims at giving an evidence-based confirmation for the mentioned assumptions. In particular, it seeks the answers to the three research questions:

1. Is there any underlying vocabulary size groups within a cohort of students who are ranked at the same vocabulary level?
2. Does the reading proficiency between students of various vocabulary groups who are ranked at the same vocabulary levels differ significantly?
3. Does the reading proficiency of learners at different vocabulary levels vary significantly within the same vocabulary group?

## 3 Methodology

The present study offers a different discovery based on the same dataset reported in Ha (2021b). In Ha (2021b), four tests were given to the same cohort of undergraduates: A LVLT, a UVLT, an IELTS listening test and an IELTS reading test. The author acknowledges that he re-analyzed the students' results on two out of the four tests administered in Ha (2021b) study: the UVLT and the IELTS reading test. As the primary objective of the paper deals with receptive vocabulary knowledge and reading comprehension, students' performance on tests of aural receptive language proficiency were not included.

### 3.1 Participants

The study analyzed data from 234 students from a highly ranked university in Vietnam. Convenience sampling was applied. All the participants were students from 8 Business English classes which the researcher was the lecturer-in-charge. Their ages ranged from 20 to 23 years old. At the time of collecting data, the participants were in the final semester of their second year at university. The native language of all participants is Vietnamese, and none of them had ever lived in any country where native English is the official language. All participants had learnt English as a second language for at least nine years and had passed three out of four obligatory business English courses at their university, generally suggesting an intermediate or B1 level of English proficiency.

### 3.2 Data collection

Data was collected in January 2021. At that time the participants just started their Business English Level 4 course. In the first week of the course, the UVLT was given to 311 students. An IELTS academic reading test was administered in the following week. However, at this stage of data collection, some students were absent, some were late and missed the tests, and some submitted their answer sheets leaving a considerable part of the reading test blank so they could leave the class early for personal purposes. Such participants were excluded from the study. Only 234 test takers satisfied the requirements for data collection. All the tests were conducted in paper-and-pencil form.

The students were well informed of the aim and importance of the study as well as the confidentiality, anonymity, and security of the collected data. They were also well aware that their performance on the tests would not affect their academic results and that they could withdraw from the study at any time. The study was reviewed and approved by University of Economics Ho Chi Minh City. All the participants provided their written consent to participate in the study.

### 3.3 Data analysis

After being collected, the students' answer sheets were graded manually and then imported in an Excel spreadsheet. At this stage, invalid data were excluded and the final set of data were then imported to SPSS 25.0 for analysis. The present study used the students' results on the IELTS academic reading test as the indicator of their reading proficiency. For approximate replication purposes, the present study utilized the research methodology applied in Laufer and Ravenhorst-Kalovski (2010) and Stæhr (2009). In particular, the way vocabulary size scores were calculated and the criteria for classifying students into vocabulary groups were identical to those applied in Laufer and Ravenhorst-Kalovski (2010). The 27/30 cut-off score used in Stæhr (2009) was also employed. Moreover, to make the analyses more updated, the study also applied the 29/30 threshold for mastery suggested in McLean (2021).

## 4 Results

### 4.1 Descriptive statistics

Table 1 reports the descriptive statistics of the participants' scores on the UVLT and the IELTS reading tests. It is clear that none of the mean scores exceeded 60% of the maximum possible scores, large standard deviations implied that the data presented sufficient spread. Reliability statistics were within the acceptable range suggested by Alavi et al., (2018), Pallant (2010), Phakiti (2016). The Shapiro-Wilk test of normality showed a normal distribution of test scores.

**Table 1:** Descriptive statistics of the IELTS listening and reading tests (N = 234).

Test	MPS	Mean	SD	Reliability
RC	40	15.86	8.377	.735
UVLT	150	86.22	26.657	.910

*Note.* RC = Reading Comprehension; MPS = Maximum Possible Score. Copyright note: the table includes data from “Exploring the relationships between various dimensions of receptive vocabulary knowledge and L2 listening and reading comprehension,” by Hung Tan Ha (2021b), *Language Testing in Asia.*

### 4.2 Vocabulary size and vocabulary level

First of all, two identical sets of data containing 234 students' answers on the UVLT and the IELTS reading test were prepared. Then, a cut-off score of 27/30, representing a 90% threshold, and a 29/30 cutting score, representing the 96.67% mastery threshold, were applied on students' UVLT scores of the first and second set of data. Participants were then ranked as “Not yet mastered any level”, “Only mastered the 1000 level”, “Mastered the 1000 and 2000 levels”, “Mastered the 1000, 2000 and 3000 levels”, “Mastered the 1000, 2000, 3000 and 4000 levels” and “Mastered all the 5 1000-word levels”. Students who reached the criterion for a lower frequency level but failed to achieve the mastery requirement for a higher frequency level were excluded from data analysis. Two students were excluded from further analysis for this reason when the 29/30 cut-off score were applied and no students got excluded for that reason for the 27/30 threshold, suggesting such an issue should not be the cause for concern when a universal cut-off score were applied.

Laufer and Ravenhorst-Kalovski’s (2010) method for estimating vocabulary score was then applied and students at each vocabulary level were then classified into different vocabulary groups according to their vocabulary size scores. The students' mean IELTS reading scores were then calculated for each vocabulary score group. Data about the number of students and their mean reading scores at each vocabulary group is illustrated in Table 2. It is interesting to see that each vocabulary level included 2–3 vocabulary groups, highlighting the fact that cut-off scores did rank students with different vocabulary knowledge as being the same vocabulary level.

**Table 2:** . Number participants of different vocabulary size and levels of mastery and their mean IELTS reading score.

Vocab level	Not yet mastered any level	Only mastered the 1000 level	Mastered the 1000 and 2000 levels	Mastered the 1000, 2000 and 3000 levels	Mastered the 1000, 2000, 3000 and 4000 levels	Mastered all the 5 1000-word levels
Vocab size						
27/30 cut-off score						
1000	N = 16 MR = 8.13 **(A1)**	N = 1	–	–	–	–
2000	N = 29 MR = 10.48 **(A2)**	N = 33 MR = 9.61 **(A3)**	–	–	–	–
3000	N = 6	N = 76 MR = 16.42 **(A4)**	N = 19 MR = 17.84 **(A6)**	–	–	–
4000	–	N = 11 MR = 21.09 **(A5)**	N = 24 MR = 22.04 **(A7)**	N = 12 MR = 24.33 **(A8)**	–	–
5000	–	–	–	N = 2	N = 3	N = 2
29/30 cut-off score						
1000	N = 17 MR = 7.82 **(B1)**	–	–	–	–	–
2000	N = 50 MR = 10.30 **(B2)**	N = 12 MR = 8.83 **(B4)**	–	–	–	–
3000	N = 26 MR = 18.42 **(B3)**	N = 71 MR = 16.72 **(B5)**	N = 4	–	–	–
4000	–	N = 29 MR = 21.41 **(B6)**	N = 15 MR = 23.33 **(B7)**	N = 1	–	–
5000	–	N = 1	N = 2	N = 2	–	N = 1

*Note*. N = number of students; MR = Mean Reading score; Vocab = vocabulary.

Since limited sample size would severely damage the generalizability of analyzed data, participant cohorts that consisted of less than 10 students were excluded from further analyses. This left the two data sets with a total of fifteen groups of participants were marked from A1 to A8 and B1 to B7 with “A” representing the 27/30 cut-off score and “B” for the 29/30 threshold.

It seems observable that reading proficiency of students of the same vocabulary size group did not differ greatly regardless of their levels of mastery. Moreover, reading scores of students at the same vocabulary level showed tremendous differences between vocabulary score groups. To confirm these speculations, eleven sets of one-way ANOVA plus Dunnett’s T3 post-hoc tests were conducted see if these differences were statistically significant. All the necessary assumptions were checked and met. Information regarding the ANOVA sets, post-hoc tests, their variables as well as the results are presented in Tables 3 and 4.

**Table 3:** One-way analysis of variance between IELTS reading test scores of different groups.

Source	SS	df	MS	F	*p*
ANOVA 1 **(A1-A2)** (Not yet mastered any level, 27/30 cut-off score)					
Between groups	57.320	1	57.320	1.171	.285
Within groups	2104.991	43	48.953		
Total	2162.311	44			
ANOVA 2 **(A3-A4-A5)** (Only mastered the 1000 level, 27/30 cut-off score)					

Between groups	1521.611	2	760.805	16.977	.000
Within groups	5243.314	117	44,0815		
Total	6764.925	119			
ANOVA 3 **(A6-A7)** (Mastered the 1000 and 2000 levels, 27/30 cut-off score)					

Between groups	187.027	1	187.027	4.146	.048
Within groups	1849.485	41	45.109		
Total	2036.512	42			
ANOVA 4 **(B1–B2–B3)** (Not yet mastered any level, 29/30 cut-off score)					

Between groups	1512.382	2	756.191	14.594	.000
Within groups	4663.317	90	51.815		
Total	6175.699	92			
ANOVA 5 **(B4–B5–B6)** (Only mastered the 1000 level, 29/30 cut-off score)					

Between groups	1370.040	2	685.020	15.802	.000
Within groups	4725.067	109	43.349		
Total	6095.107	111			
ANOVA 6 **(A2-A3)** (2000 vocabulary size, 27/30 cut-off score)					

Between groups	11.864	1	11.864	.436	.511
Within groups	1631.120	60	27.185		
Total	1642.984	61			
ANOVA 7 **(A4-A6)** (3000 vocabulary size, 27/30 cut-off score)					

Between groups	30.695	1	30.695	.499	.482
Within groups	5721.053	93	61.517		
Total	5751.747	94			
ANOVA 8 **(A5-A7-A8)** (4000 vocabulary size, 27/30 cut-off score)					

Between groups	66.785	2	33.393	1.399	.258
Within groups	1050.534	44	23.876		
Total	1117.319	46			
ANOVA 9 **(B2–B4)** (2000 vocabulary size, 29/30 cut-off score)					

Between groups	20.817	1	20.817	.770	.384
Within groups	1622.167	60	27.036		
Total	1642.984	61			
ANOVA 10 **(B3–B5)** (3000 vocabulary size, 29/30 cut-off score)					

Between groups	55.308	1	55.308	.882	.350
Within groups	5956.712	95	62.702		
Total	6012.021	96			
ANOVA 11 **(B6–B7)** (4000 vocabulary size, 29/30 cut-off score)					

Between groups	36.428	1	36.428	1.603	.212
Within groups	954.368	42	22.723		
Total	990.795	43

**Table 4:** *p*-value matrix for pairwise comparison between IELTS reading scores for different groups of students.

Vocabulary Size	1000	2000	3000	4000
**A3-A4-A5** (Only mastered the 1000 level, 27/30 cut-off score)				
2000	–	–	.000	.000
3000	–	–	–	.010
**B1–B2–B3** (Not yet mastered any level, 29/30 cut-off score)				
1000	–	.606	.001	–
2000	–	–	.001	–
**B4–B5–B6** (Only mastered the 1000 level, 29/30 cut-off score)				

2000	–	–	.000	.000
3000	–	–	–	.001
**A5-A7-A8** (4000 vocabulary size, 27/30 cut-off score)				

Vocabulary Level	Not yet mastered any level	Only mastered the 1000 level	Mastered the 1000 and 2000 levels	Mastered the 1000, 2000 and 3000 levels
Only yet mastered the 1000 level	–	–	.896	.300
Mastered the 1000 and 2000 levels	–	–	–	.553

The one-way ANOVAs and pair-wise comparisons indicated that reading scores of students of the same vocabulary groups did not differ significantly no matter whether they had or had not mastered a certain vocabulary level. Results from the analyses also showed that participants' reading proficiency varied significantly according to their vocabulary score even when they were at the same vocabulary level.

The students' reading scores between the 1000 and 2000 vocabulary size groups were not statistically different. This means that, for highly lexically demanding texts (Ha, 2021b, Table 6), learners with vocabulary knowledge below 2000 word families would similarly struggle with their comprehension, which was also a conclusion drawn by Ha (2021b).

## 5 Discussion

Firstly, the study’s findings provide supportive evidences for the hypothesized order difficulty proposed in the UVLT in particular and other BNC/COCA-based VLTs in general. In this study, less than 1% (2 out of 234) of the participants were recorded to violate the order of difficulty hypothesis by achieved the mastery requirement for lower-frequency levels but fail to meet the criteria of higher-frequency levels. In other words, the difficulty order of VLTs will be warranted as long as researchers employ a consistent cut-off score for all word levels and do not try anything different (Ha, 2021b). These results also could serve as supportive evidences for the relationship between lexical frequency and lexical difficulty.

Secondly, the present study confirmed the problem hypothesized by Ha (2021b) and Laufer (2021) regarding the ranking issue for cut-off scores when used together with VLTs. On the one hand, results from the analyses demonstrated that cut-off scores, whether strict or lenient, ranked students with different vocabulary knowledge and reading proficiency as the same level. On the other hand, reading proficiency of students from the same vocabulary score group remained consistent regardless of sample size or the mastery level of participants. The findings problematized the use of mastery thresholds in the investigation of the relationship between learners' vocabulary knowledge and language proficiency.

The research showed that vocabulary score consistently reflected learners reading proficiency, which partially supported Laufer and Aviad-Levitzky’s (2017) and Laufer’s (2021) stance on the ability of vocabulary size score to match learners to their reading level. While this may against the suggestions of some scholars (McLean, 2021; McLean and Stoeckel, 2021),I somehow believe that a student’s vocabulary score would give us a better picture of a student’s vocabulary knowledge since students tend to know some words at each band (Laufer, 2021). The general concern is that the use of cut-off scores with VLTs on groups of learners would easily underestimate the learners' vocabulary knowledge and may lead to the selection of overly easy reading materials. Personally, I believe that the cut-off scores would be especially useful when we use them to diagnose problems in the process of vocabulary learning of each individual. While the present study problematized a current practice in the field of vocabulary research, it is worth noting that the evidences it provided are not sound enough to serve as a basis for pedagogical implications. Still, teachers are encouraged to use both scoring approaches when administering VLTs to their learners. Since VLTs contain more items at each level compared to the Vocabulary Size Test (Nation and Beglar, 2007), they obviously provide a better picture of learners' vocabulary knowledge of the high and mid frequency word levels (Gyllstad et al., 2020; Stewart et al., 2021). The use of VLT combined with vocabulary score would provide teachers with a general idea of their learners' vocabulary knowledge. After that, teachers may set a particular cutting score to have more information concerning the additional support each individual student may need.

It is interesting to see that the number of participants in vocabulary score groups was strongly influenced by the students' vocabulary level (Table 2). For those students who mastered both the 1000 and 2000 levels, more than half of them were classified into the 4000-word group and no one had scores representing 2000 word families or below. The same can also be observed for other mastery levels. For example, the majority of students who “had not yet mastered any level” were placed in the 1000- and 2000-word groups and most of the participants who “had mastered only the 1000 level” were in the 3000-word group. Clearly, there seem to be a fairly linear relationship between vocabulary scores and levels. While I still hold my stance against the use of cut-off scores as an indication of learners' vocabulary knowledge, I have to admit that the relatively low level of vocabulary knowledge of the participants was indeed a limitation of the present study. A larger sample size of learners with higher levels of English proficiency would yield even more insightful views into the issue.

## 6 Conclusion

This study shed light upon the issues regarding the use of VLTs and cut-off scores and gave evidence-based confirmation to the concerns raised by Ha (2021b) and Laufer (2021). Despite being informative, the study’s power of comparison and data analysis was restrained by the generally low vocabulary knowledge of students in an EFL context. Therefore, further researches that collect larger sample size from students in both ESL and EFL contexts would yield more interesting insights into the issue.

## Declarations

### Author contribution statement

Hung Tan Ha: Conceived and designed the experiments; Performed the experiments; Analyzed and interpreted the data; Wrote the paper.

### Funding statement

This research did not receive any specific grant from funding agencies in the public, commercial, or not-for-profit sectors.

### Data availability statement

Data will be made available on request.

### Declaration of interests statement

The authors declare no conflict of interest.

### Additional information

No additional information is available for this paper.
